# Effects of eating together online on autonomic nervous system functions: a randomized, open-label, controlled preliminary study among healthy volunteers

**DOI:** 10.1186/s13030-023-00263-8

**Published:** 2023-03-10

**Authors:** Hideaki Hasuo, Nahoko Kusaka, Mutsuo Sano, Kenji Kanbara, Tomoki Kitawaki, Hiroko Sakuma, Tomoya Sakazaki, Kohei Yoshida, Hisaharu Shizuma, Hideo Araki, Motoyuki Suzuki, Satoshi Nishiguchi, Masaki Shuzo, Gaku Masuda, Kei Shimonishi, Kazuaki Kondo, Hirotada Ueda, Yuichi Nakamura

**Affiliations:** 1 Department of Psychosomatic Medicine, Kansai Medical University, Hirakata, Osaka, 573-1010 Japan; 2 Faculty of Contemporary Social Studies, Doshisha Women’s College of Liberal Arts, Kodo, Kyotanabe, Kyoto, 610-0395 Japan; 3 Faculty of Information Science and Technology, Osaka Institute of Technology, 1-79-1 Kitayama, Hirakata, Osaka, 573-0196 Japan; 4 Psychosomatic Medicine, Clinical Psychology, Faculty of Medicine, Kagawa University, 1750-1 Ikenobe, Miki, Kita, Kagawa, 761-0793 Japan; 5 Department of Mathematics, Kansai Medical University, 2-5-1 Shinmachi, Hirakata, Osaka, 573-1010 Japan; 6 Center for Research and Collaboration, Tokyo Denki University, 5 Senju, Asahi, Adachi, Tokyo, 120-8551 Japan; 7 The Section of Global Health, Department of Hygiene and Public Health, Tokyo Women’s Medical University, 8-1, Kawada, Shinjuku, Tokyo, 162-8666 Japan; 8 Academic Center for Computing Media Studies, Kyoto University, Yoshida-honmachi, Sakyo, Kyoto, 606-8501 Japan

**Keywords:** Eating together online, Eating alone, Heart rate variability, Interaction, Physiological synchrony

## Abstract

**Background:**

Eating alone has been significantly associated with psychological distress. However, there is no research that evaluates the effects or relation of eating together online to autonomic nervous system functions.

**Methods:**

This is a randomized, open-label, controlled, pilot study conducted among healthy volunteers. Participants were randomized into either an eating together online group or an eating-alone group. The effect of eating together on autonomic nervous functions was evaluated and compared with that of the control (eating alone). The primary endpoint was the change in the standard deviation of the normal-to-normal interval (SDNN) scores among heart rate variabilities (HRV) before and after eating. Physiological synchrony was investigated based on changes in the SDNN scores.

**Results:**

A total of 31 women and 25 men (mean age, 36.6 [SD = 9.9] years) were included in the study. In the comparison between the aforementioned groups, two-way analysis of variance revealed interactions between time and group on SDNN scores. SDNN scores in the eating together online group increased in the first and second halves of eating time (F[1,216], *P* < 0.001 and F[1,216], *P* = 0.022). Moreover, high correlations were observed in the changes in each pair before and during the first half of eating time as well as before and during the second half of eating time (*r* = 0.642, *P* = 0.013 and *r* = 0.579, *P* = 0.030). These were statistically significantly higher than those in the eating-alone group (*P* = 0.005 and *P* = 0.040).

**Conclusions:**

The experience of eating together online increased HRV during eating. Variations in pairs were correlated and may have induced physiological synchrony.

**Trial registration:**

The University Hospital Medical Information Network Clinical Trials Registry, UMIN000045161. Registered September 1, 2021. https://center6.umin.ac.jp/cgi-open-bin/icdr/ctr_view.cgi?recptno=R000051592.

## Background

Diet is important not only for nutritional and health aspects but also because it constitutes an essential part of daily social interactions [1]. Dietary environment can affect health from various biopsychosocial aspects. Solitary eating has been related to the development of depressive symptoms, increased mortality, and/or reduced diet quality and intake [2–5]. Recently, social isolation owing to the COVID-19 pandemic has resulted in increased solitary eating, which has been noted to be associated with psychological distress [6, 7]. Additionally, a related cohort study showed that a greater degree of unhappiness was associated with a greater proportion of eating alone [1].

Eating together has been reported to increase food intake and to improve taste through social associations with other people [8, 9]. The social facilitation of eating is defined as the promotion of an individual’s activities, such as an increase in food intake, by the presence of other people while eating [9]. Eating together is also influenced by social modeling [10], in which one’s eating behavior influences others and vice versa. As a factor affected by social interactions with other people, the feeling of relaxation enhanced by communication while eating together is important [8, 10]. Apparently, eating together increases subjective wellbeing and provides a sense of relaxation [11, 12].

In recent years, eating together online has become popular following the evolution of video conversation technologies. This phenomenon, termed digital commensality, can circumvent environmental constraints to increase the maintenance and enhancement of health from the biopsychosocial aspect of those who have been eating alone [13]. Moreover, it has been reported that eating together online can be perceived by participants as “just alone but together,” with increased food intake and reduced loneliness [14]. The results of this report suggest that eating together, even online, may stimulate social interactions. However, this has not been specifically demonstrated. Furthermore, no study has investigated the effects of eating together online on feelings of relaxation, energy or loneliness on autonomic nervous functions, which is an objective evaluation of relaxation. This study thus aimed to address these research gaps and hypothesized that eating together online affects autonomic nervous function by social interaction through a variety of factors, including feelings of relaxation, energy, and loneliness.

It has been reported that relaxation, such as by hypnosis, or intense loneliness reduces heart rate variability (HRV) at rest; reduced autonomic function can be predicted by HRV [15, 16]. It has also been reported that hand gripping between a patient with cancer and their family caregiver positively affects each other’s HRV [17]. The association or interdependency of physiological activities between two people is referred to as physiological synchrony [18]. Quantitation using maximal cross-correlation or cross-correlation with local slopes has been reported to be effective for the assessment of physiological synchrony using HRV [19, 20]. However, no method has been established yet. We thus further hypothesized that people eating together online would favorably affect each other’s autonomic functions, which would further provoke physiological synchrony.

## Methods

### Objective

This study aims to evaluate among healthy volunteers the effect on HRV of eating together online in comparison with persons eating alone.

### Study design

This is a randomized, open-label, controlled, preliminary study conducted among healthy volunteers who worked at Kansai Medical University in Osaka, Japan. The study was approved by the Medical Ethics Committee of Kansai Medical University (reference number: 2021167) and was performed in accordance with the Declaration of Helsinki (as revised in 2013). Written informed consent was obtained from all study participants before the commencement of the study procedure. The study was registered with the University Hospital Medical Information Network Clinical Trials Registry (approval number: UMIN000045161) on September 1, 2021. This study was conducted from January to April 2022.

### Study participants

The study participants were healthy volunteers, defined as “normal” persons who had no significant medical conditions or histories and no difficulty in their daily lives. They were employees at Kansai Medical University who responded to our post on the volunteer recruitment bulletin board at the university. The exclusion criteria included (1) currently taking medication or seeking medical care and (2) having neurological or mental disorders such as cognitive dysfunction and being unable to communicate. Participants were excluded if they met the diagnostic criteria for neurological or mental disorders according to the fifth edition of the Diagnostic and Statistical Manual of Mental Disorders [21], confirmed by two psychosomatic physicians.

### Study procedures

**Fig. 1:**
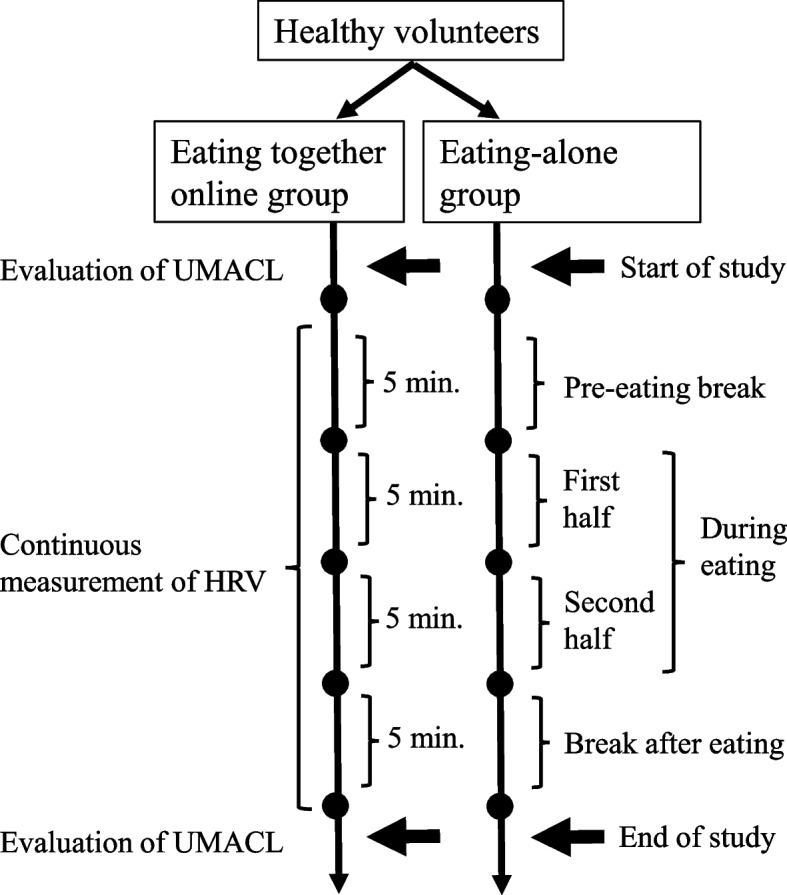
Flowchart of the study procedures. UMACL, UWIST Mood Adjective Checklist; HRV, Heart rate variability

Figure 1 summarizes the study regimen. Participants were randomized into either an eating together online (pairing) group or a control (eating alone) group by a computer using the minimization method at a 1:1 ratio. Each participant was informed of their allocated group after randomization. The participants were not allowed to change their groups during the study period. Concurrently, the investigators participating in the study were also informed of their designated groups. The study staff in charge of the statistical analysis concealed the results of the randomization. The participants’ names were also kept anonymous. The data were collected in interview rooms by the clinicians responsible for the study. Each investigator interviewed and assessed the participants at the beginning of the study.

The study participants completed a self-report questionnaire, the UWIST Mood Adjective Checklist (UMACL), at the beginning and end of the study period. Each study subject attached a special electrode pad connected to the HRV measurement device (myBeat WHS-1; Union Tool Co., Tokyo, Japan) to their chest. The participants ate a snack for 10 min and took breaks for 5 min each before and after eating. The 10 min eating time was divided into first and second halves. The data evaluated using the HRV were continuously recorded for 20 min. The HRV scores for each session were calculated based on the mean of the 5-min HRV record. We used HRV analysis software (Kubios HRV version 3.1; Kubios Oy, Kuopio, Finland), which is highly reliable for short-term recording [22].

The snacks were commercial cookies. The participants eating together online ate the snacks while looking at their partner on the computer screen, during which conversation was allowed. The participants in the eating-alone group ate the snack while looking at an offline black screen, during which conversation was not allowed; however, soliloquy was allowed. During the 20-min eating time, including snack time and the short breaks, participants remained in their allocated eating rooms.

### Evaluation methods

#### UWIST mood adjective checklist

The Japanese version of the UMACL was used to evaluate the mood of comfort [23]. The original checklist, developed by Matthews et al., was created based on dimension theory, making it possible to assess arousal levels [24]. The scale has two subscales that can be used to evaluate energetic arousal (10 items; vigorous vs. tired: coefficient α = 0.79) and tense arousal (10 items; nervous vs. relaxed: coefficient α = 0.76) [25]. High energetic arousal represents active and happy, whereas low tense arousal represents calm and quiet. Participants were asked to respond on a 4-point Likert scale. In a previous study, the mean energetic arousal was 24.4 (standard deviation [SD]: 0.5) for males and 24.4 (SD: 0.4) for females, and the mean tense arousal was 18.5 (SD: 0.4) for males and 17.56 (SD: 0.3) for females [25].

#### Heart rate variability and standard deviation of the normal-to-normal interval

HRV, the fluctuation of heartbeat intervals measured using an electrocardiogram, is used to evaluate autonomic nerve activities [26, 27]. HRV tends to be lower in a person with anxiety or depression. However, it is relative rather than absolute; therefore, it is not directly compared among individuals.

Standard deviation of the normal- to- normal interval (SDNN) is the quantification of HRV to further compare it among individuals. Particularly, SDNN is the standard deviation of the R-R intervals of the heartbeat in a certain time duration and is obtained via time-domain analysis. SDNN was used to evaluate cardiovascular compatibility. SDNN includes all the different types of variations and represents total variability [28]. It assesses the flexibility of the autonomic nervous system and the balance of sympathetic and parasympathetic nervous systems, with an increase in SDNN reflecting the stability of these systems [16, 29]. The grand mean of SDNN scores among resting adults is 50 mseconds [29].

### Endpoints

The primary endpoint of this study was the change in the SDNN score before and during eating. The key secondary endpoints were the amount of cookie intake, change in UMACL score, and correlation coefficient of pairs in change in SDNN score.

### Sample size estimation

This is a preliminary study conducted among healthy volunteers to evaluate the effects of eating together online on autonomic nervous system functions. To the best of our knowledge, no similarly designed studies have been conducted previously. Therefore, we recruited as many study volunteers as possible.

### Statistical analysis

Continuous data are summarized as means with SD, and discrete data are presented as the number of subjects (n) and their frequencies (%), as appropriate. Pearson’s chi-square test was used to evaluate discrete data, including age, sex, and mutual relationships. An unpaired two-sided t-test was used to compare mean age. Changes in UMACL scores (before and after eating) were analyzed using one-way repeated-measures analysis of variance (ANOVA). To compare the change in the UMACL and SDNN scores between the two groups, two-way repeated measures ANOVA with fixed effects of time points and groups was used. Moreover, the variable effect of subjects was used to examine the time course changes of these scores. In ANOVA, multiple comparisons were corrected using Bonferroni’s method. Lastly, after calculating the correlation coefficients of pairs in the change in SDNN scores, we performed Fisher’s z-transformation on the correlation coefficients followed by the z-test statistic.

The last UMACL and SDNN scores of participants who withdrew from the study before completion were used for analysis. A significance level of alpha < 0.05 was used for statistical analysis. Statistical analyses were conducted using SPSS version 18.0 J for Macintosh (SPSS Inc., Chicago, IL, USA). Only the z-test statistic was calculated based on web links and references without using SPSS [30, 31].

## Results

### Clinical demographic characteristics

A total of 56 healthy volunteers were randomized into either the eating together online group (*n* = 28) or the eating-alone group (*n* = 28) or 28 pairs (100.0%) and completed the study. Table 1 presents the demographic and clinical characteristics of the participants. The mean age was 36.6 years (SD: 10.1), and 25 were male and 31 female. The mutual relationships of the participants included 47 work colleagues and nine friends. There were no group differences in age, sex, or mutual relationship.

**Table 1:** Demographic and mutual relationships of the study participants

	Eating together online group		Eating-alone group		
	(*n* = 28)		(*n* = 28)		*P*-value
Age (year), mean (SD)	37.1	(10.3)	36.1	(9.9)	0.711
Sex, n (%)					
Male	11	(39.3)	14	(50.0)	0.296
Female	17	(60.7)	14	(50.0)	
Mutual relationships					
Work colleague	23	(82.1)	24	(85.7)	0.500
Friend	5	(17.9)	4	(14.3)	

*SD* Standard deviation

#### Primary outcome analysis

### Changes in SDNN scores and between-group comparisons

**Fig. 2:**
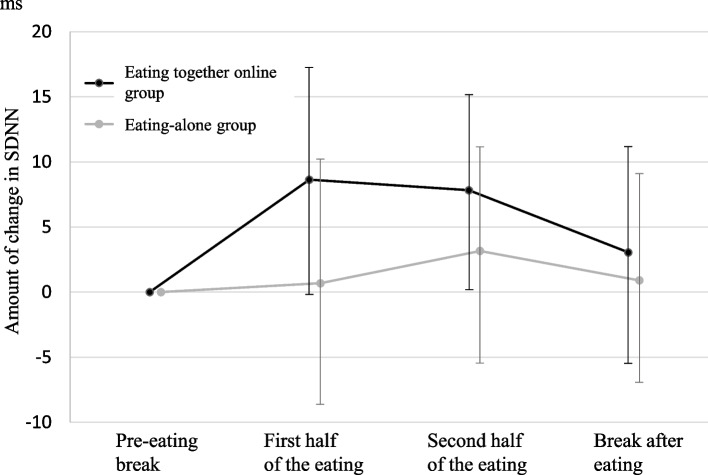
Changes in SDNN between the pre-eating break and during or break after eating. SDNN, standard deviation of the normal-to-normal interval

Figure 2 shows the changes in the SDNN scores between the pre-eating break and during eating or the break after eating. During the pre-eating break, the SDNN scores were 32.8 (SD: 10.8) and 32.2 (SD: 12.7) for the eating together online and eating-alone groups, respectively. The SDNN scores for the eating together online group were significantly higher during eating than during the pre-eating break, rather than thereafter (first half of eating; *P* < 0.001, second half of eating; *P* < 0.001, rest after eating; *P* = 0.071). The SDNN scores for the eating-alone group were higher during and after eating than during the pre-eating break. However, the difference was insignificant (first half of eating; *P* = 0.708, second half of eating; *P* = 0.093, rest after eating; *P* = 0.556).

A two-way ANOVA showed an interaction between time and group in the change in SDNN score (F(3,216), *P* = 0.037). The change in SDNN score of the eating together online group was significantly higher than that of the eating-alone group before and during the first half of eating, and before as well as during the second half of eating (F[1,216], *P* < 0.001 and F[1,216], *P* = 0.022). However, the difference did not differ for before and after the breaks (F[1,216)]; *P* = 0.287).

#### Secondary outcome analysis

### Amount of cookie intake

The amount of cookie intake was 4.2 (SD: 2.6) for the online eating together group and 3.5 (SD: 2.5) for the eating-alone group (*P* = 0.310).

### Changes and between-group comparisons of scores

Table 2 shows the change in the UMACL scores and comparisons between the groups. The mean energetic arousal scores at the beginning of the study were 30.0 (SD: 1.2) for male and 31.3 (SD: 0.5) for female participants, while those for mean tense arousal were 26.9 (SD: 0.5) and 26.3 (SD: 0.3), respectively. The energetic arousal score was higher after eating in the eating together online group and differed between the groups (*P* = 0.036). The tense arousal score was lower after eating in both the groups, with no difference between the groups (*P* = 0.199).

**Table 2:** Changes and between-group comparisons of UMACL scores

	Eating together online group					Eating-alone group					*P*-value
	Before eating		After eating		*P*-value	Before eating		After eating		*P*-value	
	mean	SD	mean	SD		mean	SD	mean	SD		
Energetic Arousal score	30.5	3.7	32.4	3.4	0.001	30.7	3.4	29.9	3.7	0.284	0.036
Tense Arousal score	26.8	1.5	24.6	1.7	0.001	26.7	1.2	25.9	1.5	0.034	0.199

*SD* Standard deviation*, UMACL* UWIST Mood Adjective Checklist

### The correlation coefficients of pairs in changes in SDNN scores

**Fig. 3:**
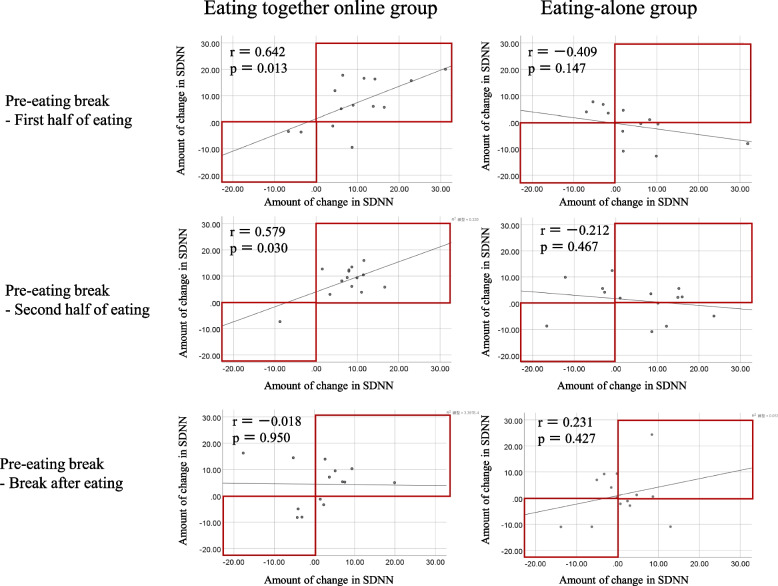
The correlation coefficients of pairs (r) in changes in SDNN scores. SDNN, standard deviation of the normal-to-normal interval

Figure 3 shows a plot of changes in the SDNN scores for each pair. Comparing the correlation coefficients of both groups revealed that the eating together online group had higher correlations of pairs in changes both before and during the first half of eating and before and during the second half of eating; these differed significantly compared with the eating-alone group (*P* = 0.005 and *P* = 0.040). Before the break and during the first half of eating in the eating together online group, 10 out of 14 pairs showed positive changes in the SDNN score. However, two pairs showed negative changes. Before the break and during the second half of eating in the eating together online group, 13 out of the 14 pairs showed positive changes in the SDNN score. However, one pair showed negative changes. Nevertheless, the two groups differed insignificantly before and after the breaks (*P* = 0.405).

## Discussion

To the best of our knowledge, this is the first study to examine the effects of eating together online on reciprocal HRV using objective parameters.

The first important finding in this study is that eating together online significantly increased HRV during eating, compared with eating alone, implying that eating together online enhanced autonomic functions during eating [26, 27]. This is a favorable outcome for people who are not able to eat together, such as those who are admitted to facilities or hospitals or physically separated from their families. During the recent COVID-19 pandemic, hospitalized patients with cancer have been prohibited from receiving visitors. Consequently, more than half of them reported feeling lonely [32]; further encouragement of eating together online is desirable.

The UMACL reported that eating together online resulted in significantly higher energetic arousal postprandial than pre-snacking, indicating that vigorous arousal may affect autonomic function. Tense arousal decreased in both groups, and there was no significant difference. The mean tense arousal score, a subscale of UMACL, in this study was significantly higher than that in a previous study [25], and it is possible that significant differences between before and after the study were difficult to obtain. SDNN assesses the flexibility of the autonomic nervous system and the balance of sympathetic and parasympathetic nervous systems [16, 29]; hence, it is likely to be affected by various factors such as energetic arousal, tense arousal, and loneliness. Another study reported that significant loneliness reduces HRV [16]. Therefore, our result regarding increased HRV following eating together online may indicate alleviated loneliness associated with the presence of other people. Apparently, autonomic signals, such as electrocardiogram and electrodermal activity, were synchronized with each other because the strangers were in the same space without direct communication [33]. Future studies using self-administered questionnaires of loneliness will be useful for investigating whether visual presence in the same space, even online, reduces loneliness.

The second important finding of this study is that the pre- and intra-snack correlations of SDNN changes when eating as a pair online were statistically significantly stronger compared with those eating alone. This suggests that eating together online may positively affect HRV through social interaction with other people. Notably, during the second half of eating in the eating together online group, the SDNN changes were synchronized in all pairs, including one pair whose SDNN scores were both negative. This suggests that the physiological synchrony of eating together online may have increased as the diet progressed. Methods to assess physiological synchrony in eating together have not yet been established. Therefore, we implemented a new method to visually clarify that physiological synchrony of change in SDNN occurs in each pair only in the eating together online group.

Although the design of our study does not identify the cause of this effect on social interactions, energetic arousal may have contributed to the outcome of the UMACL.Apparently, pairing with a person with a poor relationship is more likely to increase physiological synchrony as assessed by HRV than pairing with a friend [19]. The report stated that this may be because if relationships with others were poor, participants tried to increase affinity to establish social affiliation. We believe that increasing affinity may also increase energy arousal. More than 80% of the participants in this study were work colleagues; however, poor relationships may have influenced social interactions and energetic arousal.

Lastly, unlike previous studies [8, 9], there was no difference in ingestion between the two groups in our study. Previous studies state that the increased ingestion for eating together online groups is attributed to choosing and eating more types of food owing to social interactions with other people [8]. However, this study was one type of cookie only.

This study has two limitations. The first is that in the eating-alone group, the element of conversation was missing in the assessment of its effects on autonomic nervous system functioning during eating. Future studies creating an eating together online group that does not talk would be able to reduce this bias. Second, the results of the study cannot be generalized because it was conducted among relatively young, healthy volunteers who were work colleagues employed by the same institution. Sociodemographic characteristics such as being prone to eating alone, male sex, older age, and unemployment are listed [1]. Work colleagues were particularly difficult to assess because of their varying degrees of relationship. Finally, no structured interviews were conducted to screen participants for neurological or mental disorders such as depression and anxiety.

## Conclusions

The experience of eating together online increased HRV during eating. Variations in pairs were correlated and may have induced physiological synchrony.

## Data Availability

The datasets analyzed in the current study are available from the corresponding author upon reasonable request.

## Abbreviations

HRV: Heart rate variability
SDNN: Standard deviation of the normal-to-normal interval
UMACL: UWIST Mood Adjective Checklist
SD: Standard deviation
ANOVA: Analysis of variance
